# Risk for non Hodgkin’s lymphoma in the vicinity of French municipal solid waste incinerators

**DOI:** 10.1186/1476-069X-7-51

**Published:** 2008-10-29

**Authors:** Jean-François Viel, Côme Daniau, Sarah Goria, Pascal Fabre, Perrine de Crouy-Chanel, Erik-André Sauleau, Pascal Empereur-Bissonnet

**Affiliations:** 1 CNRS n° 6249 "Chrono-Environment", Faculty of Medicine, 2, place Saint Jacques, 25030 Besançon cedex, France; 2 National Institute for Public Health Surveillance, "Health and Environment" Department, 12, rue du Val d'Osne, 94415 Saint-Maurice Cedex, France; 3 Haut-Rhin Cancer Registry, and French network of cancer registries (FRANCIM), 87, avenue d'Altkirch, 68051 Mulhouse, France

## Abstract

**Background:**

Dioxin emissions from municipal solid waste incinerators are one of the major sources of dioxins and therefore are an exposure source of public concern. There is growing epidemiologic evidence of an increased risk for non-Hodgkin's lymphoma (NHL) in the vicinity of some municipal solid waste incinerators with high dioxin emission levels. The purpose of this study was to examine this association on a larger population scale.

**Methods:**

The study area consisted of four French administrative departments, comprising a total of 2270 block groups. NHL cases that had been diagnosed during the period 1990–1999, and were aged 15 years and over, were considered. Each case was assigned a block group by residential address geocoding. Atmospheric Dispersion Model System software was used to estimate immissions in the surroundings of 13 incinerators which operated in the study area. Then, cumulative ground-level dioxin concentrations were calculated for each block group. Poisson multiple regression models, incorporating penalized regression splines to control for covariates and dealing with Poisson overdispersion, were used. Five confounding factors were considered: population density, urbanisation, socio-economic level, airborne traffic pollution, and industrial pollution.

**Results:**

A total of 3974 NHL incident cases was observed (2147 among males, and 1827 among females) during the 1990–1999 time period. A statistically significant relationship was found at the block group level between risk for NHL and dioxin exposure, with a relative risk (RR) of 1.120 (95% confidence interval [CI] 1.002 – 1.251) for persons living in highly exposed census blocks compared to those living in slightly exposed block groups. Population density appeared positively linked both to risk for NHL and dioxin exposure. Subgroup multivariate analyses per gender yielded a significant RR for females only (RR = 1.178, 95% CI 1.013 – 1.369).

**Conclusion:**

This study, in line with previous results obtained in the vicinity of the incinerator located in Besançon (France), adds further evidence to the link between NHL incidence and exposure to dioxins emitted by municipal solid waste incinerators. However, the findings of this study cannot be extrapolated to current incinerators, which emit lower amounts of pollutants.

## Background

France has the largest number of municipal solid waste incinerators (MSWIs) in the European Union, with 128 plants counted in 2006. Their technology has evolved over time, with a general reduction of emissions that affect nearby communities. Thus, at present, all MSWIs in France meet the European guide value (0.1 ng/m^3^). However, older units have undeniably caused significant pollution in the past. This has contributed throughout the years to an overall increase in the environmental load of particulate matter that contains dioxins, metals, and accumulates in soils and local food.

Dioxin emissions from MSWIs are one of the major sources of dioxins and therefore are an exposure source of public concern. According to the International Agency for Research on Cancer, one of the dioxin congeners, the 2,3,7,8-tetrachlorodibenzo-*p*-dioxin (TCDD), is carcinogenic to humans [1]. In this assessment, human carcinogenicity data mainly came from cohort studies of industrial populations. Nevertheless, many controversial issues related to this subject still persist, especially regarding their impact on public health [2,3]. Scholars continue to debate whether low environmental doses of dioxins affect the general population, particularly in the vicinity of an MSWI [4]. Other pollutants emitted by incinerators might also be involved, including heavy metals, polycyclic aromatic hydrocarbons (PAHs), and dust.

The etiology of the most common non-Hodgkin's lymphoma (NHL) types remains elusive, and the only well-established risk factors (immune-deficiency disorders, autoimmune diseases, some viruses in specific subentities) explain a small percentage of NHL occurrence [5]. However, increased incidence of, and mortality from NHL have been reported in several investigations conducted on cohorts of workers exposed to TCCD, thus suggesting a role for dioxin in NHL development [6-10]. Several case-control studies also found an association between exposure to dioxin contaminated pesticides and NHL [11]. Moreover, the epidemic of NHL observed during the second half of the 20^th ^century has now started to level off in North America and Europe [12]. This time trend might be partly explained by one or several environmental agents (including dioxins) with decreasing exposure of the population (because of regulations enforced in the 1970s and 1980s) [13].

Recent evidence regarding NHL and MSWI has progressed in France from sequential studies that started with crude investigations, and were gradually refined to more specific, targeted studies carried out around a MSWI with high emission levels of dioxins (Besançon). A cluster of NHL was first detected in an area that contains the MSWI [14]. Subsequently, a 2.3-fold risk (95% confidence interval [CI], 1.4 -3.8) for NHL was associated with residence in areas classified as highly exposed to dioxins emitted from this MSWI [15]. In parallel, the validity of the geographic-based exposure categories was assessed through dioxin measurements from soil samples [16].

These results prompted the national authorities to develop a nationwide study to analyze the relation between cancer risk and past exposure to MSWIs among neighbouring populations. This paper focuses on NHL risk.

## Methods

### Study area

The study took place in four French administrative departments (Isère, Bas-Rhin, Haut-Rhin, Tarn), which were covered by a population based cancer registry (part of an international network of such registries). These registers are complete for NHL as ascertained by the ratio of the number of deaths to the number of cases registered during 1993–1997 (ranging from 39 to 50%, and from 42 to 69% among males and females, respectively), very similar to those reported in other western countries [17]. Virtually, all cases were histologically verified (98 to 99%). The study area comprised a total of 2270 communities (census block groups with a relatively homogenous population of approximately 2000 inhabitants), used as statistical units.

### Cancer data

The cases considered for this study were aged 15 years and over, who had been diagnosed with NHL during the period 1990–1999, and were living in the study area at the time of their diagnosis. Cancer registries extracted anonymous data on date of birth, gender, date of diagnosis, address at the time of diagnosis, and cancer category (using the International Classification of Disease for Oncology, second edition, morphology codes M9590-9595/3, M9670-9723/3, and M9761/3). Cases were assigned to a block group by residential address geocoding (with a georeferencing rate of 99%). Ethical approval was granted in 2005 by the National Commission for the Confidentiality of Computerized Data (n° 05-1171).

### Inventory of MSWIs

The examined exposure period ranged from 1972 to 1985 (to allow a mean 10 year latency period), as a function of emission dates for the 13 incinerators that operated in the study area for at least one year during this period. Very few past measurements of emissions (total particulate matter, dioxins, or metals) were available, mainly from 1996 onwards. We had therefore to rely on expert assessments to estimate exposure from the 13 incinerators in the same way. First, operators and local public authorities were asked for technical descriptions of each plant. These included variables known to influence dioxin emission: capacity (four categories: < 1 ton/h, 1–3 ton/h, 3–6 ton/h, > 6 ton/h), type of functioning (continuous, discontinuous), dust control (present, absent), fume treatment (present, absent), and year operations began. Then, six experts, representing operators (4), and research institutions (2), were invited to estimate concentration of exhaust gas (in ng/Nm^3 ^or mg/Nm^3^) for each pollutant (dioxins, furans and PCBs -hereafter called "dioxins"-, carcinogenic metals – As, Cd, Cr VI, Ni -, and particulate matter of diameter less than 10 μm [PM10]). To apply an iterative process towards consensus (the Delphi method), the experts were each independently asked to provide an estimate, and to discuss discrepancies until they reached a consensus for each of the 13 MSWIs, and each pollutant. Finally, concentration estimates were transformed into emission flow rates (in ng/s or mg/s), assuming that one ton of waste produced 6000 Nm^3 ^of fumes in the 90s (generation factor used by the European Commission [18]).

### Atmospheric diffusion modelling

A second-generation Gaussian atmospheric diffusion model (Atmospheric Dispersion Model System version 3 – ADMS 3) was used to compute "immission" estimates (immission is the amount of pollutant reaching a particular location as a result of – and in contrast to – the emission coming out the chimney) for each category of pollutants (dioxins, metals, dusts) in the surroundings of MSWIs (from 5 to 20 km, depending on the incinerator) [19]. The dispersion modelling methodology used a receptor grid spacing of 200 m. Parameters considered were the quantitative estimates obtained above from the experts, chimney height and diameter, plume emission temperature, particle size and density, topographic indicators (roughness, relief), local weather data, etc. The latter came from the national meteorological agency (Météo France). Immission estimates served as proxies for annual ambient air concentrations of chemicals attributable to the MSWI at a given location.

### Exposure of communities (block groups)

Population exposure to pollutants emitted by MSWIs was estimated only as a function of the geographic zone of residence. The immission estimate assigned to each of the 2270 communities was the median of all receptor estimates located in each block group (the mean block group surface was 9.45 km^2^, corresponding on average to 231 receptors). When a community was under the plume of several MSWIs, the exposure index was defined as the sum of the estimated indexes of each. We calculated two different exposure indexes for each community, according to different hypotheses about the mode of exposure: (1) the immission estimate alone represented exposure from inhalation only (expressed in ng/m^3^); (2) this score was transformed to account for the number of years the plant had operated and the degradation speed in soils, yielding an average cumulative ground-level concentration since the start of the activity (expressed in μg/m^2^/year).

**Figure 1:**
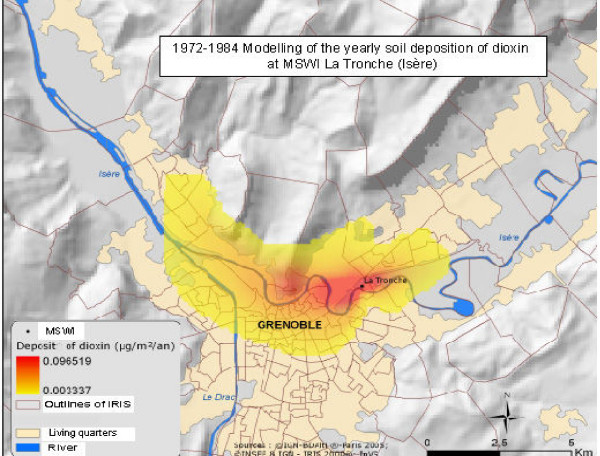
Example of ground-level concentration modelling in the vicinity of a municipal solid waste incinerator (La Tonche, Isère, France).

We focused on dioxins (since the other pollutant emissions or immissions were highly correlated with dioxin ones), and average cumulative ground-level concentrations (that showed a dose-response relationship with disease risk). Among the 2 270 block groups, 520 laid within modelled zones (Figure 1). Non zero value (the minimum modelled ground-level concentration under a plume, i.e. 1.85 × 10^-5 ^μg/m^2^/year) was ascribed to block groups outside a MSWI plume influence.

A rich set of social and demographic information was available for every block group. Five possible confounding factors mentioned in the literature could therefore be extracted: population density (inhabitants/km^2^), urban or rural character of the place of residence (based on the French Census Bureau urban-rural continuum code: urban, unipolar suburban, multipolar suburban, and rural), socio-economic level (ad hoc deprivation index built with census data), airborne traffic pollution (with nitrogen dioxide [NO_2_] pollution from on-road vehicles, expressed in μg/m^3^, as a proxy), and industrial pollution (estimated through polluting industry × years weighted by block group surface).

### Statistical analysis

Units considered in the statistical analyses consisted of the 2270 block groups.

Expected numbers of cases for each block group were computed by applying reference incidence rates for the same years (as estimated from the four administrative departments of the study area, plus the departments of Doubs and Hérault) to the person-years of each area stratified by gender and 5-year age classes.

Models were fitted to the grouped data with Poisson regression analysis. Logarithms of observed and expected cases were linked with a set covariate values in a linear model that dealt with Poisson overdispersion. To build flexible models, penalized regression splines (based on 25 knots) were used [20]. Since the dioxin concentration distribution was right-skewed, a square root transformation was applied to force the exposure data to follow a normal distribution. Log-transformed industry × years, log-transformed population density, socio-economic level, and NO_2 _concentration were entered as continuous variables in the models. Urbanisation (yes/no, by collapsing the 3 urban categories), and administrative departments (department of Isère as reference category) were treated as categorical variables.

Multivariate models were run. Dioxin exposure (variable under scrutiny) and administrative departments (to account for their heterogeneity) were forced into the models. Then, the 5 remaining independent variables were introduced as spline functions. To assess the smoothing added value, we followed the ad hoc approach proposed by Wood et al. [20]. If the estimated degrees of freedom were close to 1 (and a linear function was therefore estimated), and if the generalized cross validation (GCV) score was higher than the GCV for the unsmoothed model, then the original unsmoothed variable was preferred to the spline-smoothed version. Finally, using Akaike's information criterion for covariate selection, a backward stepwise selection was applied.

Hierarchical Bayesian Poisson regression models were also fitted to these geographical data to address the issue of spatial autocorrelation [21].

**Figure 2:**
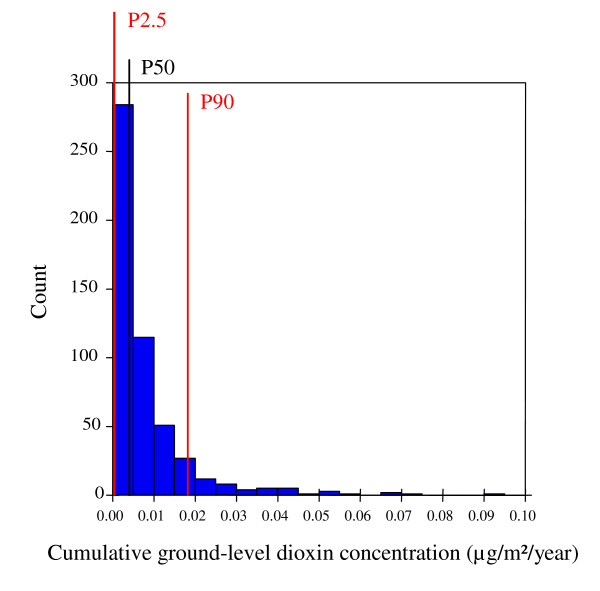
**Dioxin exposure (modelled ground-level concentrations) distribution within modelled zones (520 block groups, France).** P2.5 = 2.5^th ^percentile (1.25 × 10^-4 ^μg/m^2^/year); P50 = 50^th ^percentile (4.25 × 10^-3 ^μg/m^2^/year); P90 = 90^th ^percentile (1.78 × 10^-2 ^μg/m^2^/year).

The relative risk (RR) associated with dioxin exposure was therefore computed as $\text{RR}=\text{exp}\left(\hat{\beta }\left(\sqrt{{\text{x}}_{\text{2}}}-\sqrt{{\text{x}}_{\text{1}}}\right)\right)$. Under a high exposure scenario, we have a priori defined x_1 _(the reference concentration) as the 2.5^th ^percentile (1.25 × 10^-4 ^μg/m^2^/year), and x_2 _as the 90^th ^percentile (1.78 × 10^-2 ^μg/m^2^/year) of the dioxin exposure distribution within the modelled zones (Figure 2).

Models were run with R software (package mgcv) and WinBUGS software [22,23].

## Results

During the 1990–1999 time period, a total of 3974 NHL incident cases was observed: 2147 among males (mean age: 61.49 years, standard deviation [sd]: 16.21 years), and 1827 among females (mean age: 66.06 years, sd: 16.44 years). A detailed analysis highlighted a standardised incidence ratio (SIR) heterogeneity between departments (Table 1).

**Table 1:** Non Hodgkin's lymphoma cases and standardised incidence ratios (1990–1999), and populations (1995) in four French administrative departments

	Male				Female			
Department	Observed cases	Expected cases	SIR*	Population	Observed cases	Expected cases	SIR	Population

Isère	727	716.9	1.01	411,569	597	585.7	1.02	432,797
Bas-Rhin	711	654.8	1.09	386,256	622	571.9	1.09	415,826
Haut-Rhin	476	469.5	1.01	268,989	395	403.9	0.98	285,384
Tarn	233	313.5	0.74	137,661	213	259.2	0.82	148,792
Total	2147	2154.7	1.00	1,204,475	1827	1820.7	1.00	1,282,799

* standardised incidence ratio

A statistically significant relationship was found at the block group level between risk for NHL and dioxin exposure, in both a univariate analysis (*p*-value = 10^-5^), and a multivariate analysis (*p*-value = 0.04) (Table 2). The best goodness-of-fit was obtained with a model including three confounding factors (departments, log(industry × years), and spline-smoothed log(population density) with 1.963 effective degrees of freedom. In the first rounds of backward stepwise iterations, the introduction of population density heavily decreased the strength of the association with dioxin exposure (results not shown).

**Table 2:** Association of dioxin exposure (modelled ground-level concentrations) with non-Hodgkin's lymphoma incidence (3974 cases, four administrative departments, 1990–1999, France)

	Regression coefficient	Standard deviation	*P*-value
	*Univariate analysis*		
Dioxin exposure*	1.453	0.328	10^-5^
	*Multivariate analysis*		
Dioxin exposure	0.925	0.459	0.04
Department			
Isère^†^	-	-	-
Bas-Rhin	0.094	0.042	0.03
Haut Rhin	0.008	0.046	0.87
Tarn	-0.180	0.058	0.002
Log(industry × years)	0.022	0.011	0.05
S(log(population density))^‡^	-	-	0.07

* modelled ground-level concentrations

^† ^reference category

^‡ ^spline-smoothed log(population density) with 1.963 effective degrees of freedom.

Neither a regression coefficient nor a standard deviation can be reported, as for any spline curve (divided by definition into many segments corresponding to the knots).

**Figure 3:**
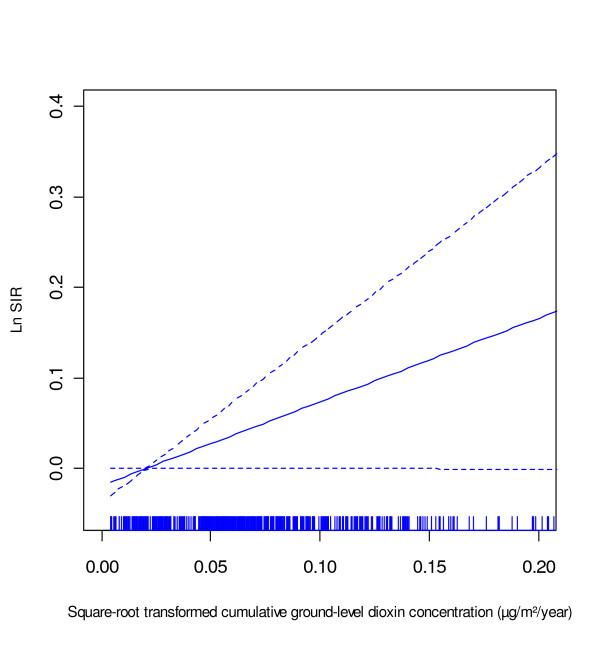
Log-transformed SIR for non-Hodgkin's lymphoma vs. square root transformed cumulative ground-level dioxin concentration (μg/m^2^/year) (1990–1999, France).

Figure 3 displays log-transformed SIR for NHL vs. square root transformed dioxin concentration, while adjusting for departments, log(industry × years), and spline-smoothed log(population density). A clear positive and linear trend is noticeable.

RR for persons living in highly exposed census blocks compared with those living in the slightly exposed block groups (90^th ^percentile/2.5^th ^percentile) was 1.120 (95% CI 1.002 – 1.251).

Subgroup analyses per gender are reported in Tables 3 and 4. Unsurprisingly, best fitting models were different. Among males, modelled ground-level dioxin concentrations revealed significant in the univariate model (*p*-value = 0.04), but not in the multivariate approach (Table 3).

**Table 3:** Association of dioxin exposure (modelled ground-level concentrations) with non-Hodgkin's lymphoma incidence among males (2147 cases, four administrative departments, 1990–1999, France)

	Regression coefficient	Standard deviation	*P*-value
	*Univariate analysis*		
Dioxin exposure*	0.954	0.464	0.04
	*Multivariate analysis*		
Dioxin exposure	0.106	0.625	0.86
Department			
Isère^†^	-	-	-
Bas-Rhin	0.116	0.056	0.04
Haut Rhin	0.065	0.065	0.32
Tarn	-0.177	0.097	0.07
Airborne traffic pollution	0.008	0.005	0.08

* modelled ground-level concentrations

^† ^reference category

**Table 4:** Association of dioxin exposure (modelled ground-level concentrations) with non-Hodgkin's lymphoma incidence among females (1827 cases, four administrative departments, 1990–1999, France)

	Regression coefficient	Standard deviation	*P*-value
	*Univariate analysis*		
Dioxin exposure*	1.978	0.442	10^-5^
	*Multivariate analysis*		
Dioxin exposure	1.340	0.628	0.03
Department			
Isère^†^	-	-	-
Bas-Rhin	0.102	0.060	0.09
Haut Rhin	-0.008	0.066	0.91
Tarn	-0.049	0.085	0.56
Log(industry × years)	0.038	0.016	0.01
Log(population density)	0.023	0.015	0.14
Urbanisation	0.182	0.078	0.02

* modelled ground-level concentrations

^† ^reference category

Conversely, RRs appeared statistically significant among females, both in the univariate (*p*-value = 10^-5^) and the multivariate (*p*-value = 0.04) models, yielding a RR of 1.178 (95% CI 1.013 – 1.369) for the latter.

No extra Poisson variation was highlighted with the hierarchical Bayesian Poisson regression for any model (results not shown).

## Discussion

This study highlights a statistical association between residence under the plume of a MSWI and an increased risk for NHL during the 1990s.

The strengths of this study are fourfold. First, this study used a population-based design. Cases were actively identified through multiple sources within defined geographic areas and benefited from a very high georeferencing rate. Second, this study used sophisticated modelling of emissions as a proxy for dioxin exposure. The modelled ground-level concentrations represented the best available surrogates for past dioxin exposure measurements given that no earlier measurements (in soils or blood of nearby residents) had been taken. Third, owing to the fine geographical level used, this study was able to allocate a different exposure to NHL cases living in the same town, but not in the same block group, thus making an exposure misclassification less likely (but we had to assume that residents within a given contour were homogeneously exposed). Fourth, the total number of cases (3974) ensured a sufficient statistical power to highlight a moderate excess risk.

Our methodology also presented some limitations. The validity or appropriateness of the dispersion models have not been assessed (and had to be assumed over time in this study of long-term effects), because we lacked the necessary household and soil measurements in the contaminated areas. Residence location as a surrogate of exposure cannot distinguish contributions from the direct and the indirect exposure pathways (e.g., from air to soil and home-grown produce). Moreover pollutant-specific deposition models (dioxin, metals, and dusts) are interrelated and thus pollutant effects are difficult to parse. However, the estimate of dioxin emissions should not be considered as a tracer for the whole mixture, since experts specifically assessed dioxin emissions. Finally, it was impossible to determine whether cases developed NHL in the same block group where they were diagnosed. Considering the long exposure-to-effect interval, in- and out migrations may have occurred, inducing a potential non-differential misclassification.

This is an ecologic study, that does not deal with individual subjects or individual-level traits or exposures, but rather with the characteristics of block groups. Although some relevant confounding factors were considered at the block group level (socioeconomic status, population density, etc.), we lacked individual information pertaining to residence history, occupational history, food consumption, etc., that could potentially confound the relationship between dioxin exposure from the municipal solid waste incinerator and NHL. Thus, we cannot firmly exclude the possibility that residual confounding affected the reported regression coefficients.

We found a significant association among females but not among males. It has been shown that the male-to-female incidence rate ratios are greater than 2 for high grade or peripheral T-cell NHL (constituting, however, only about 17% of all NHL) [24]. The risk for NHL among men versus women may also vary by site. A predominance of NHL regional to the respiratory tract among men, and the retroperitoneum among women, has been reported in a Swedish study [25]. Risk factors (e.g. environmental or occupational hazards) are therefore probably different for men and might blur an association with environmental dioxins [25]. Future epidemiological studies, at the individual level, should deal with this gender issue to check its consistency.

A number of studies have reported a significant positive association between western world cancer incidence and urban areas (compared to their counterparts in rural areas) or population density [26]. Some authors argue that as population density increases so too does economic output and the by-products of that economic activity, possible pollutant factors [27]. In our study, population density was positively linked both to risk for NHL and dioxin exposure. It could represent a broad indicator for a number of possibly interconnected factors (personal health behaviours, socio-economic status, viruses, psycho-social stress factors, environmental pollutions, etc.). Exposure to some of these urban-associated risk factors, occurring more commonly among females than among males, could explain the gender disparity observed in our results.

Five possible confounders were considered, among which airborne traffic and industrial pollutions representing potential sources of the same pollutants under study. They could therefore challenge the accuracy of exposure assessment. However, they only marginally affected the regression coefficient estimates (results not shown).

Our study is in line with previous results obtained in the vicinity of another French MSWI [14,15]. However, direct comparisons cannot be made, since the latter emitted higher dioxin levels and has been studied at a finer geographical level (blocks), with a different (case-control) design. Conversely, Biggeri et al. did find a localized cluster of deaths for NHL in the surrounding area of a MSWI in Campi Bisenzio (Tuscany, Italy), but among males [28].

Results provided by Bertazzi et al. on the 20-year mortality of the Seveso population are of utmost importance, because they bridge the gap between occupational and environmental exposure levels. As a point of clarification, people in the Seveso cohort had mean TCDD blood lipid concentration of 136 ng TCDD/kg, which falls between the typical occupational dioxin levels (> 1,000 ng TCDD/kg) and background levels (2–3 ng TCDD/kg) [29]. Allowing for a latency time window of 15–20 years, results for NHL clearly did stand out, according to Bertazzi et al., with a relative risk of 2.8 (95% CI = 1.1–7.0) [30,31]. These results have recently been confirmed in an extended follow-up study (25 years) [32].

Recently, Baccarelli et al. proposed a biological mechanism that could explain the dioxin-NHL association [33]. More than 80–90% of follicular and 20% of diffuse large lymphomas carry the t(14;18) translocation, the most frequent chromosomal translocation in human lymphoid malignancies. They found that TCDD exposure was related to increased numbers of t(14–18)-positive circulating lymphocytes, but not to the proportion of t(14;18)-positive subjects, in healthy individuals from Seveso, suggesting that TCDD may promote clonal expansion of non-malignant cells carrying t(14;18).

## Conclusion

This study adds further evidence to the link between NHL incidence and exposure to dioxins emitted by MSWIs. However, the findings of this study cannot be extrapolated to current MSWIs, which emit lower amounts of pollutants and are more closely controlled than past MSWIs. Future large-scale population based studies that include histological grading, assessment of nutritional, residential and occupational history, and dioxin blood measurements are needed to lead to new insights into the association between environmental dioxin exposure and NHL.

## Abbreviations

ADMS3: Atmospheric Dispersion Model System version 3; CI: Confidence Interval; I-TEQ: International Toxic Equivalency factor; MSWI: Municipal Solid Waste Incinerator; NO_2_: Nitrogen Dioxide; NHL: Non-Hodgkin's Lymphoma; OR: Odds Ratio; PM10: Particulate Matter of diameter less than 10 μm; PCBs: Polychlorinated Biphenyls; PAHs: Polycyclic Aromatic Hydrocarbons; RR: Relative Risk; TCDD: 2,3,7,8-tetrachlorodibenzo-*p*-dioxin.

## Competing interests

The authors declare that they have no competing interests.

## Authors' contributions

JFV participated in the study conception and design, and prepared the manuscript. CD carried out the atmospheric diffusion modeling and the exposure quantification. SG performed statistical analysis and provided statistical expertise. PF organized data collection procedures, and managed daily operations of field team. PCC oversaw the GIS part of the research. EAS contributed with academic discussions and revisited the manuscript. PEB participated in the study conception and design, and supervised the study. All authors read and approved the final manuscript.
